# Finite Element Model Updating of RC Bridge Structure with Static Load Testing: A Case Study of Vietnamese ThiThac Bridge in Coastal and Marine Environment

**DOI:** 10.3390/s22228884

**Published:** 2022-11-17

**Authors:** Duc Cong Nguyen, Marek Salamak, Andrzej Katunin, Michael Gerges

**Affiliations:** 1 Department of Mechanics and Bridges, Faculty of Civil Engineering, Silesian University of Technology, Akademicka 5, 44-100 Gliwice, Poland; 2 Department of Fundamentals of Machinery Design, Faculty of Mechanical Engineering, Silesian University of Technology, Konarskiego 18A, 44-100 Gliwice, Poland; 3 Faculty of Science and Engineering, University of Wolverhampton, Alan Turing Building, Wulfruna Street, Wolverhampton WV1 1LY, UK

**Keywords:** RC bridge, FE model update, GA optimisation, structural health monitoring, SOFISTIK TEDDY

## Abstract

Diagnostic load testing refers to the use of the measured historical responses of the structure in the field data to better understand its dynamic and static structural behaviours. It is important and necessary to predict the health state, load capacity, and aging of the structure by updating the finite element (FE) model, which can give useful information to aid the design of retrofits and the maintenance of the existing bridge in the future. The paper presents an update of the full-scale FE model for the reinforced concrete (RC) bridge structure over the seawater river based on the experimental strains under the static load testing in which the representative FE model of the actual structure is determined from the optimisation procedures. The optimisation variables are applied, including the cross-sectional properties and concrete material calibrated through the genetic algorithm (GA) optimisation in the MATLAB software, which interfaces with the FE modelling in the scripting of the SOFISTIK TEDDY software automatically. The bending moments at the mid-span of the RC girders are determined in the FE modelling to compute stresses, which are compared with the measured stresses through optimisation scenarios with a percentage error of the objective function less than 10%. The measured data of concrete strains are recorded from reusable strain transducers installed on the mid-span girders for every bridge span, which are used to calibrate the bridge model in static load testing. The novelty of the solution is to implement innovative techniques using field data as an improved approach for calibrating automatically the analytical FE model parameters of all RC spans of the bridge until its static behaviours are very similar to those of the actual bridge. The final updated FE modelling is used to apply truck load configurations according to bridge design standards such as the AASHTO specifications, which can predict the load limits of the existing bridge structure more accurately and reliably. These proposed approaches can be applied to large bridges as well as complex structures with supporting FE analysis software and data processing software.

## 1. Introduction

The new technological trends of field structural evaluation techniques have been widely applied in various engineering sectors, in which commercial applications of bridge evaluation have developed around three major aspects, including non-destructive evaluation (NDE) methods, controlled load tests and structural health monitoring (SHM) [1]. As an example, diagnostic load tests of bridge structures have been used efficiently to predict load limits by updating the FE model based on design codes and guidelines to help manage existing bridges, which will aid decisions on the maintenance and replacement of bridges [2]. In another SHM study of a cable-stayed bridge, the SHM of the structure was conducted to track the time–strain history responses of the bridge under dynamic vehicle passes. Other interesting studies on intelligent SHM systems of structures have been implemented as automatic alarms and smartphone-based applications [3,4,5]. The FE model updating of the existing bridge structure based on data sets from the SHM system has been the key important goal of the innovative solution to determine the representative FE model of the actual structural responses, for the estimation of load limits according to design codes and standards, as well as for predicting the current health state of the existing structures.

Excellent examples of full-scale FE model updating based on field-measured and computed data were studied to modify the local element stiffness properties of structural members of existing bridges for evaluation and load rating [6,7,8,9,10,11]. Similarly, the historical dynamic and static behaviours of the existing bridge were investigated by the updated FE model, which confirmed that the design standards were applied to the load rating [12]. A few other studies performed FE model calibration techniques of bridges using experimental modal analysis, in which the slave and master stiffness parameters were adjusted by GA and PSO methods [13,14,15,16,17]. Moreover, another example of updating structural behaviours under static loads and dynamics was presented through SOFISTIK for the pedestrian drawbridge across the Motlawa River in the city of Gdansk [18]. A much more detailed discussion of the FE model updating was based on sensitivity analysis [19,20]. The papers focused on the FE mode updating using field data sets with a wide range of novel improved approaches, including GA and PSO optimisation methods, implemented efficiently in the representative model of the structure. The main purposes of these applications are to optimise the stiffness properties of structural members in the existing bridges based on the vibration analysis of accelerometers in the field testing, but there is little industrial research and innovation based on the FE model updating of the field strain behaviours for the bridge structures. In recent times, modern bridges have been built with new technologies and materials, in which the updating of the structures has been investigated to modify the stiffness parameters based on the field experimental modal analysis, as with the studies introduced above. However, traditional bridge structures have presented obstacles and significant challenges when applying these approaches. The FE model update of the existing RC bridges using the historical field responses of intelligent strain transducers in the SHM system has been the best way to deal with this problem.

In order to achieve the more accurate and reliable FE model update of the structure, novel innovative approaches have been developed to interface between the finite element analysis (FEA) software and other powerful optimisation tools in the MATLAB and PYTHON software in some recent papers. Several investigators have applied the optimisation techniques by interfacing the MATLAB software with the ANSYS, SAP2000 and ABAQUS software, which are powerful and user-friendly tools to automatically update the FE models of structures [21,22,23]. One other example of the FE model updating of a bridge based on the strain responses of the model was achieved using the ANSYS software [24]. Some other techniques for the FE model updating of the steel bridge were developed in the SAP2000 and SOFISTIK software [25]. Moreover, an interesting explanation was provided of the optimisation procedure of the geometric parameters of the arch bridge by connecting the Autodesk REVIT software and the PYTHON software in the Dynamo software [26]. Some FEA software has been built into the FE model of structures with useful features added, including a parametric model for geometry, analysis and design, as well as improvements implementing design codes and standards, in which the FE model calibration based on the field experimental data sets has been the most complex optimisation procedure when only the functions and features available in the software are used.

Improved approaches have been developed to interface between the FE model generated in the FEA SOFISTIK software and robust optimisation algorithms in the MATLAB software, implemented with functions to automatically modify the material and cross-sectional properties of structural members and components using the historical strain responses of the bridge; these factors highlight the potential weaknesses of the previous studies in terms of bridge inspection and assessment. The final calibrated FE models of the representative structures integrated with bridge design codes and checks in bridge safety and evaluation procedures have not been fully discussed in recent papers; another gap relates to the key important roles of full-scale FE model updating for the strategic planning of the repair, replacement, upgrading and life-cycle maintenance of existing civil structures.

The proposed case study concerns the FE model updating of the existing bridge with the GA optimisation technique based on the measured strains under static load testing, so that the representative FE model of the actual structure can be determined by the improved optimisation approach. The scripts and functions in MATLAB contain an optimisation algorithm that interfaces with the CADINP language script with the FE modelling of the bridge structure implemented in SOFISTIK TEDDY to automatically update the stiffness variables. The test truck will be applied to the FE modelling as 2D load points, where the numerical strains in the mid-span at the bottom of the concrete girders are determined and compared with the measured strains. The bending moments at the mid-span of the RC girders will be computed to determine the numerical stresses of the middle cross-sections, which will be used to calibrate the measured data of the bridge under the static load testing. The proposed approaches will be applied to the Vietnamese RC bridge under static truck load testing based on the historical responses in the field, and the final updated FE model of the existing bridge structure will be used for load ratings according to bridge design codes and standards. The final updated FE models of spans will be utilised to apply the load configurations according to current design standards and codes, in which recommendations for improving the load capacity and serviceability of the existing RC bridge are provided to ensure that it remains in service or is repaired.

## 2. Review of Field Instruments for Non-Destructive Evaluation and SHM Systems of Bridge Structures in Vietnam

**Figure 1:**
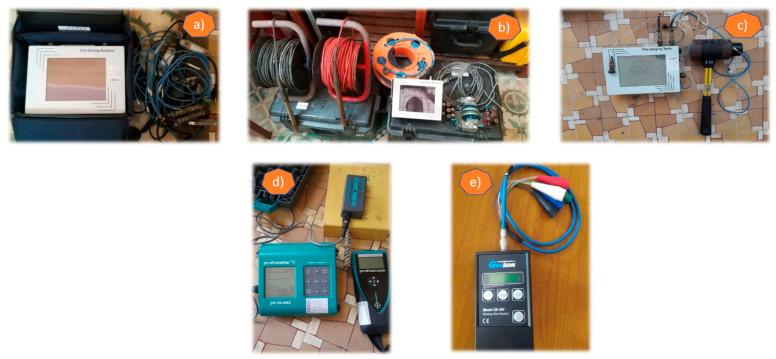
Field equipment used in NDE methods and SHM techniques on bridge structures: (**a**) Pile driving analyser system; (**b**) Cross-hole analyser system; (**c**) Pile integrity tester system; (**d**) Proceq Profometer device; (**e**) Geokon readings.

Various instruments and technologies have been used for the non-destructive evaluation (NDE) and structural health monitoring of bridge structures, as shown in Figure 1.

(a) The pile driving analyser (PDA) system performs the high strain dynamic load testing on the deep foundations, such as the piers, abutments, pilots and piles, of bridges and buildings, which can evaluate the foundation capacity, shaft integrity and driving stresses based on the accelerometers and strain transducers.
(b) The cross-hole analyser (CHA) system evaluates the concrete quality of the drilled shafts and the cast-in-place concrete piles in the deep foundations using the cross-hole sonic logging (CSL) method.
(c) The pile integrity tester (PIT) system reveals cracks, necks and voids in concrete piles by pulse echo methods.
(d) The Proceq Profometer device detects the locations of the rebars and performs the measurement of the concrete cover and the steel-reinforced bars embedded in the bridge structures.
(e) The Geokon readings are used for the strain measurements in concrete foundations, piles, bridges, dams, tunnels and buildings by embedding the vibrating wire strain gauges in large aggregate concrete structures.

**Figure 2:**
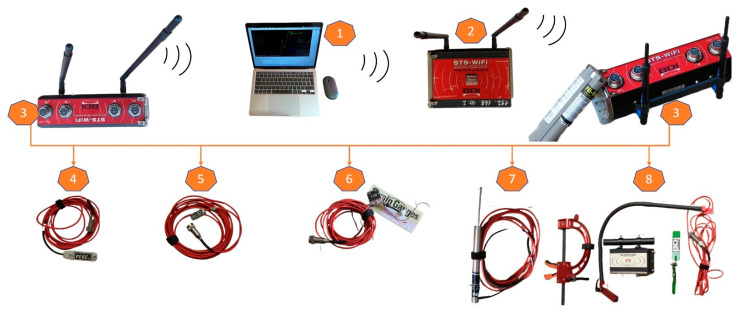
The wireless structural testing system for field diagnostic load testing of bridge structures: (1) WinSTS software in laptop’s Windows; (2) Mobile-based WiFi station; (3) Four-channel WiFi Node; (4) Intelligent strain transducer; (5) MEMS-based accelerometer; (6) 350 Ω foil gage amplified completion module; (7) Linear Variable Differential Transformer (LVDT) displacement transducer; (8) Auto-clicker device.

The wireless structural testing system (STS-WiFi) of Bridge Diagnostics Inc., from Louisville, CO, the USA, has been widely used for the field load testing of different types of existing structures to implement various sensors, including intelligent strain transducers, accelerometers, strain gauges, LVDT displacement sensors and auto-clickers, as shown in Figure 2.

(1) The WinSTS data acquisition software can control the WiFi data acquisition hardware nodes and the WiFi mobile-based station to record field data from the sensors. It can display the state of every node, such as the power, signal strength, name, standby mode or ‘sleep’ function. The monitoring sensors operate in real time so that one can set up zero sensors and access the calibrated sensor file. The sample rate, test duration and data file name can be assigned to collect data.
(2) The mobile-based battery-powered WiFi hardware station can directly communicate with the WinSTS data acquisition software, which can control more than one WiFi data acquisition hardware node, also connected by an ethernet Internet cable through four ethernet ports and WiFi.
(3) The four-channel WiFi data acquisition hardware node is powered by a rechargeable battery, using wireless technology to communicate with the WiFi mobile-based hardware station, which communicates wirelessly with a laptop and iPad for a signal range of more than 1.0 km. This WiFi node system can implement a wide variety of sensors.
(4) Intelligent strain transducers are installed in steel members and reinforced concrete structures.
(5) Accelerometers record the dynamic behaviour of structures and concrete piers.
(6) The micro-strain measurements are integrated with reusable quarter bridge foil strain gauges, which can measure the strain of the different materials, e.g., fibre-reinforced polymer (FRP), reinforced steel bars.
(7) The LVDT displacement sensors are used to determine the deflection of structural members and spans.
(8) The auto-clicker is used to track the position of moving trucks at every wheel revolution, and it is placed on the driver-side front wheel.

## 3. A Case Study: Vietnamese ThiThac Bridge

### 3.1. Optimisation Approach

**Figure 3:**
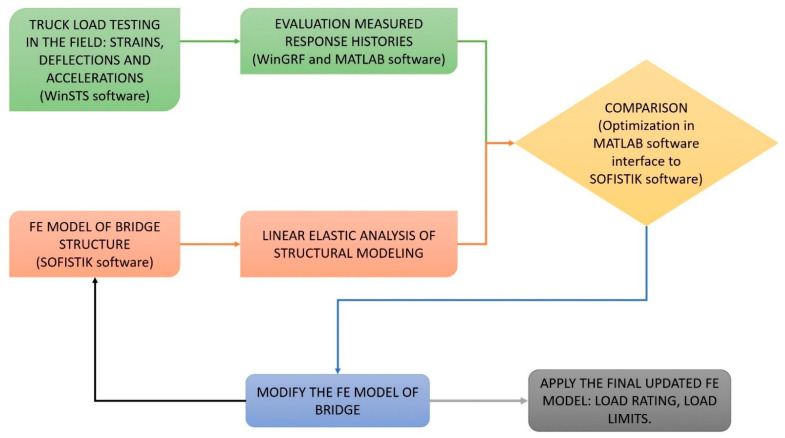
Approach of FE model updating using SOFISTIK and MATLAB software.

The load testing of the existing bridge refers to the field-measured data to better understand the behaviour of the structure under static load vehicles in the linear elastic range. Note that the type of field testing will provide primarily information about the position of the test truck for the bridge, and not its load limit capacity. The capacity of structural members can be calculated based on the design standards and material properties, which can be combined with the measured data and the analytical values in the updated FE model to predict load limits and structural damage. The goal of the FE model updating is to implement techniques to modify the parameters of the FE model of the bridge; we provide a diagram of this approach in Figure 3. The illustration provides a practical outline for implementing the GA optimisation algorithm in the MATLAB software to interface with the FE model in the SOFISTIK TEDDY software. In the following, we outline the procedures:

(1) Run the static load test on the bridge using WinSTS software;
(2) Evaluate and assess the measured data using WinGRF software and MATLAB software;
(3) Generate and analyse the linear elastic FE model of the structure using SOFISTIK software;
(4) Compare the measured strain responses with the numerical strain results of the FE model until the modelling errors are minimised;
(5) Apply the load ratings using design standards and codes in the final updated FE model, and then assess other problems such as structural damages from the final adjusted FE model.

Load ratings based on the updated FE model for the existing bridges generally have more benefits in that structural behaviours can be fully recorded by the FE method. The truck loads can be positioned in the full-scale FE model according to the design codes and standards of the structures.

Moreover, one of the key innovative approaches is the optimisation process, which should be automated by connecting the numerical results from the SOFISTIK software with the robust optimisation algorithms in the MATLAB software to control the input stiffness parameters of the FE bridge modelling. There are three main procedures in which the integrated approach has been used to modify the analytical FE model stiffness parameters of the bridge structure to obtain behaviour similar to that of the actual bridge structure based on the static strain responses in the field testing; then, the results of the final calibrated FE model can be considered for use for load rating, permit load and other predictions via the bridge design codes and standards. This proposed optimisation approach, based on the experimental responses of the bridge structure in field testing, has been used to evaluate the structural health, as well as the load rating, in order to be similar to the bridge design optimisation methods according to the bridge design specifications that can be found in the literature, in line with the detailed examples cited above, including different data input, output and objective functions. More detailed step-by-step procedures and algorithms using static strain responses are as follows:

(1) The first step in generating the realistic FE bridge model in the SOFISTIK software using the TEDDY text editor and the CADINP input language is to simulate the planar geometry of the bridge model. This includes beam elements (BEAM NO) to represent concrete beams and diaphragms, shell/plate elements (QUAD) for the concrete deck slab and spring elements (SPRI NO) for the elastic restraints of support boundary conditions. The geometry (SECT) and stiffness properties (MAT) are defined for concrete bridge beams and concrete deck slabs, as well as elastic spring supports. The stiffness parameters of the material and the cross-sectional properties of the structural members can be added by the command lines as #DEFINE, so that the possible input file is generated with the input file name as bridge.dat or bridge.txt, implemented in the SOFISTIK software, and the new parameters are obtained after every iteration step.
(2) The second step involves developing the command lines in the MATLAB software to read the input file to interface with the SOFISTIK software, so as to analyse the FE bridge model automatically and connect it with the computational results of the FE bridge model. For example, the command lines in the MATLAB software are the following:
  > ga(error function, number of paramerters, [], [], [], [], lower bound, upper bound, [], [], optimoptions(‘ga’));
  > system(sprintf(‘ ”%s” %s ’, “…\SOFiSTiK 2022\sps.exe”, input file)).
(3) The third step involves evaluating the final updated FE bridge model with the new stiffness parameters after each iteration. The load cases are modelled in the SOFISTIK software by using the command lines as LC and POI AUTO TYPE, and the numerical stress and strain behaviour of the bridge structure is analysed and saved as output files as strain.csv, ratingfactor.csv, etc. This procedure is the same as in the first step. Stopping criteria should be applied, in which the average percentage error of the objective function is less than 10% and additional error values between experimental and numerical strain responses should be also less than 10%.

The optimisation error equation of the objective function can be expressed as follows.

(1) $$g\left(z\right)=E_{strain}=\frac{1}{n}\overset{n}{\underset{i=1}{\sum }}{\left|\frac{\varepsilon_{i}^{m}-\varepsilon_{i}^{c}}{\varepsilon_{i}^{m}}\right|}^{2}$$

where *E_strain_* is the error function of the experimental and numerical strains; different squared values are divided by the experimental strains squared; n represents the number of measured response histories; $\varepsilon^{m}$ is the measured strain response in the field testing; $\varepsilon^{c}$ is the computational strain in the FE model.

The following *N* master and slave variables’ objective functions can be summarised as

(2) $$\begin{array}{c} \;\;Minimize\;\;\;\;\;\;\;\;\;\;\;\;\;\;\;\;\;g\left(z\right)=g\left(z_{1},z,\ldots ,z_{N}\right)\\ Subject\;to\;\;\;\;\;z_{i}^{ll}\;\leq \;z_{i}\;\leq \;z{\;}_{i}^{ul},\;\;\;i=1,2\ldots ,N \end{array}\}$$

where $z_{i}^{ll}$ and $z_{i}^{ul}$ represent the lower and upper limits, respectively.

The load limits of existing bridges are evaluated through the final updated FE modelling, in which the numerical results of the longitudinal stresses of the structural members are determined by using the automatic output of the SOFISTIK software. The load rating procedure is based on the Allowable Stress (AS) method according to the American Association of State Highway and Transportation Officials (AASHTO). The AASHTO load configurations are applied in the updated FE model, in which the analytical bending stress of each structural member is calculated step by step from the maximum flexural moment after every optimisation process. The rating factor (*RF*) for moment stresses is calculated as [27,28]

(3) $$RF=\frac{\sigma_{xx}^{C}-\sigma_{xx}^{DL}}{\sigma_{xx}^{LL}\;\left(1+IM\right)}$$

where $\sigma_{xx}^{C}$ is the allowable stress capacity of structural members in flexure; $\sigma_{xx}^{DL}$ is the maximum flexural stress due to a dead load; $\sigma_{xx}^{LL}$ is the maximum flexural stress due to a live load; *IM* is the impact factor with the live load effect (AASHTO or measurement).

The approach based on the final calibrated FE model for the load rating of the existing bridges is composed of four main steps:

(1) Apply the design load standards to the final FE model;
(2) Compute the predictions of the stress levels of the key structural members;
(3) Perform the load rating calculation using the *RF* equation;
(4) Check if *RF* ≥ 1 for the bridge, which passes the design loads, or if *RF* < 1, it fails the legal vehicle loads.

The rating of the bridge (*RT*) in tons for the structural member of the diagnostic load test, if its *RF* is less than 1.0, is as follows [29]:

(4) RT=(RF) W

where *W* is the weight in tons of the nominal truck load according to the design codes and standards.

**Figure 4:**
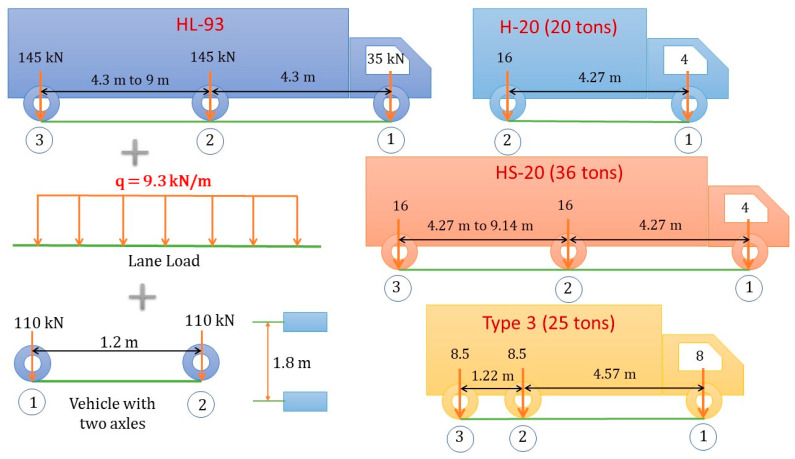
Bridge loading truck configurations according to AASHTO design standards.

The main purpose of the FE model updating is to obtain a more realistic model of the bridge in which the load configurations of the common vehicles according to AASHTO design codes including H-20, HS-20, Type 3, Type 3S2 or Type 3-3 to the structure for design load rating and permit load limits will be applied. Furthermore, the loading scenarios related to the positions, the gross weight of the truck in each group of axles and the distances between the groups must be defined in the calibrated FE modelling of the bridge structure, as described in Figure 4. The actual load test for the existing bridge in the field could be different from that used for legal AASHTO vehicles. When the span length is very short, so that it cannot apply all standard design loads, some vehicles should be considered if the following AASHTO requirements are met. As an example for this study, the four structural spans are short, so some load configurations could be implemented in the FE modelling, such as HL-93, H-20, HS-20 and Type 3.

### 3.2. Structure Description and Field Test Procedure

The Vietnamese ThiThac bridge is a four-simple-span reinforced concrete (RC) beam and deck bridge that has an asphalt pavement layer surface and guardrails. The bridge is located on the old national highway over the saltwater river that flows into ocean water. The lengths of spans 1, 3 and 4 are 9.1 m, while the length of span 2 is 8.05 m. The widths of the roadway and the structure are 8.62 m and 9.58 m, respectively. The thickness of the asphalt roadway deck is 5 cm. The thickness of the concrete deck ranges from 6 to 10 cm in the centre. The ThiThac consists of eight rectangular RC girders with height 0.4 m and width 1.18 m. The girder spacing is 1.2 m. Static load tests were performed by a two-axle dump truck across the bridge according to three truck paths, including the centric position and the eccentric position on the right and left sides. The truck tested (78C02978) had a total gross weight of 8.42 tons, a weight of 3.58 tons and a weight of 4.84 tons in the rear. The distance between the front and rear axles was 5.6 m, while the spacing between two wheels was 1.87 m.

**Figure 5:**
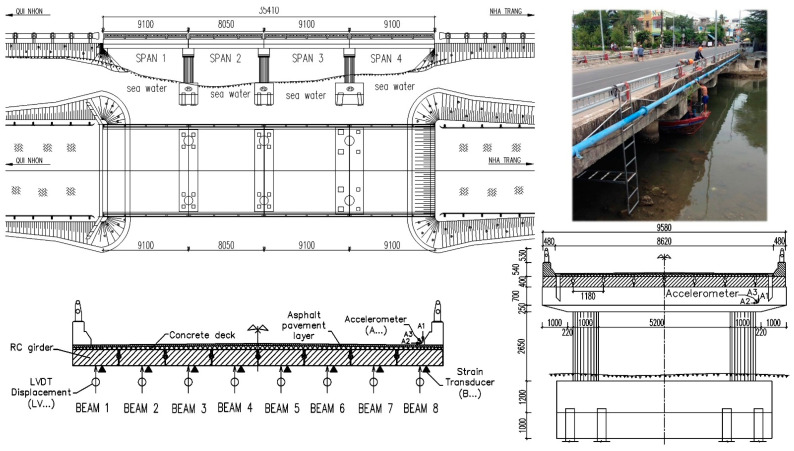
Overview of the Vietnamese ThiThac bridge and instrumentation plan.

The goal of the instrumentation plan was to measure the static responses of structural girder members and to record the dynamic behaviour of the bridge. The bridge was instrumented with reusable intelligent strain transducers and accelerometers for each span, as shown in Figure 5. The structural testing system of this bridge structure in field testing uses the mobile base station with antennas to connect with many four-channel nodes that implement different types of weatherproof sensors, including intelligent strain transducers (full wheatstone bridge with 350 Ω foil gages, ±4000 µε, effective gauge length with 76.2 mm); LVDT displacements (±75 mm); and accelerometers (±5 g). Each four-channel STS-WiFi node can connect to the mobile base station by connecting the wireless network and can communicate wirelessly with the user’s laptop. WinSTS software is used to collect field data sets in real time by connecting to the STS-WiFi system, with sample rates from 0.1 Hz to 500 Hz (max), as well as automatically zero before the test. The battery-powered mobile base station and rechargeable battery-powered nodes can use the CAT5E Ethernet cable to extend the range of the Internet connection with the communication protocol interface (802.11 b/g). The battery life of the mobile base station is approximately 8 h, while the battery life of the nodes is approximately 6 h. The auxiliary power of the mobile base station and nodes can be incorporated with the 110–220 V AC power adapter to convert the 12 V DC output to operate without batteries for the long-term structural health monitoring of infrastructures in an indeterminate time. The range of WiFi signals between the node and mobile base station can be several hundred metres and can be extended by adding mobile base stations up to 1.0 km.

The instrumentation and testing procedures of the bridge structure were performed with the main plans, including the following:

(1) Install intelligent strain transducers attached to the eight concrete beams on the surface located at the middle bridge span near the bottom of the cross-sectional centroid to measure the static strain responses, in order to determine the axial force of each structural member. Bridge span 1 was installed with five strain transducers, while bridge spans 2, 3 and 4 were instrumented with eight strain transducers in each structural span. Some bridge spans could be installed, and the LVDT displacement sensors only measured the deflection responses of two spans (1 and 4) near the area of the bridge abutment where the foundation of the scaffold system could be placed. Accelerometers were attached to the top deck and each pier and abutment to measure the dynamic responses of each structural bridge span in the vertical, horizontal and longitudinal directions under the high-speed vehicle. Attachment methods of strain transducers and accelerometers on the bridge structure include C-clamps, threaded mounting tabs and quick-setting adhesive, wood screws or concrete anchors, installed in a non-destructive manner, which can be removed easily after field testing.
(2) Data sets in field testing were recorded with three load cases (left and right eccentric positions, centric load) of static load testing (sampling frequency recommended 30 Hz–80 Hz or better), one load case of dynamic testing (sampling frequency over 100 Hz to 250 Hz) with a high-speed truck at 100 km/h and three truck paths travelling at 5 km/h for quasi-static load testing (sampling frequency less than 50 Hz). Every load case test cycle was run three times to ensure reproducibility in the data files.
(3) The WinSTS data acquisition software is used as the computer interface for the STS-WiFi hardware under a Windows operating system environment, which can control all functions in the STS-WiFi equipment to collect data. It can serve all main functions, including outputting a real-time graphical display; sensors’ calibration factors; auto zero mode; and providing detailed information on mobile base stations, nodes and sensors. One of the most important steps is to set the sampling frequency and reset all sensors to zero values before testing.

### 3.3. Experimental Results of Field Load Testing

**Figure 6:**
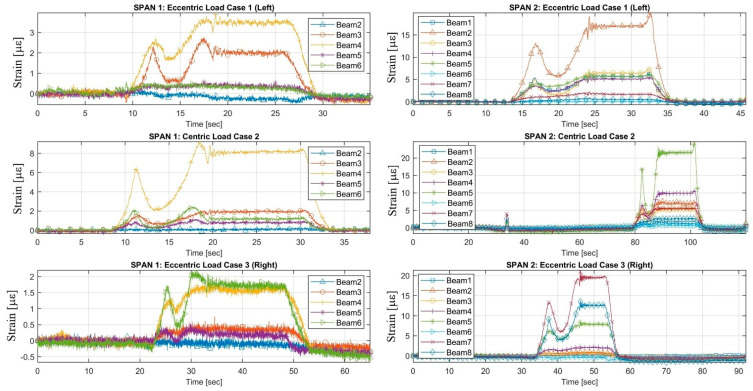
The results of measured strain responses for bridge spans 1 and 2.

**Figure 7:**
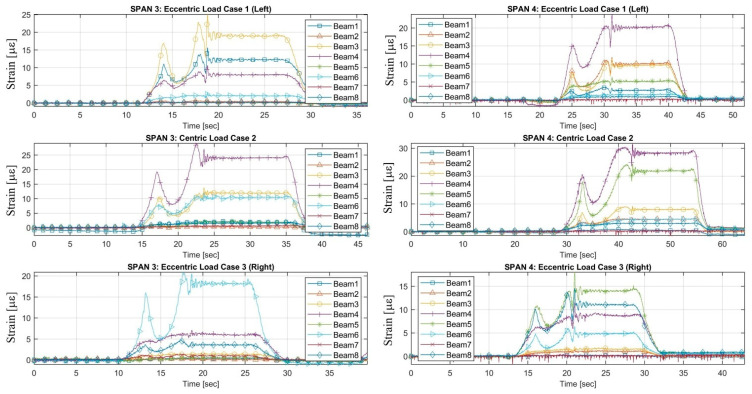
The results of experimental strain responses for bridge spans 3 and 4.

**Figure 8:**
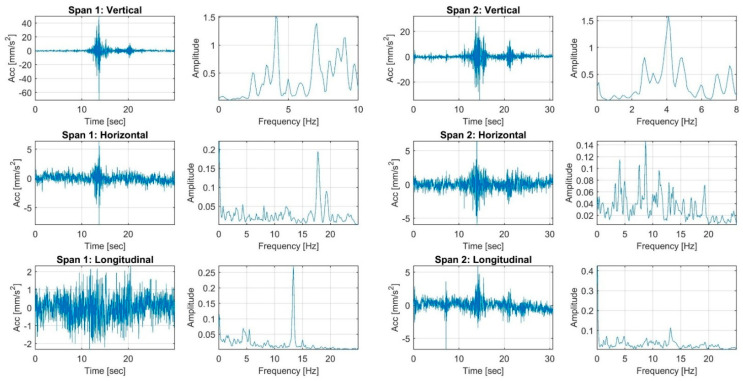
The high-speed dynamic FFT results of accelerometers for spans 1 and 2.

**Figure 9:**
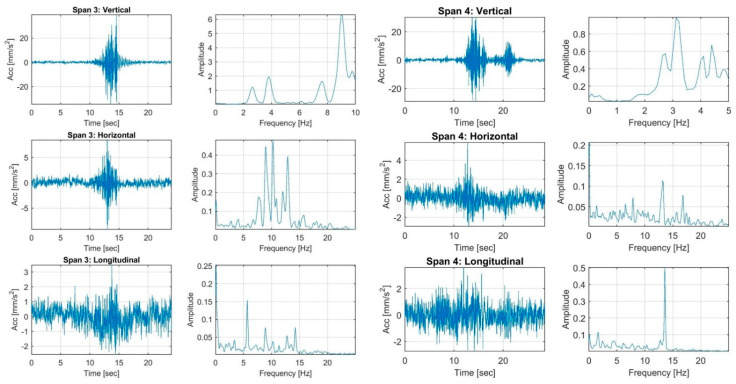
The high-speed dynamic FFT results of the accelerometers for spans 3 and 4.

The load tests were performed by the single truck applied to the Vietnamese ThiThac RC bridge structure as concentrated vertical centric and eccentric loads. The strain histories of the RC girder members under three static load cases, including a centric load and eccentric load on the left and right sides of the centre line of the bridge, are shown in Figure 6 and Figure 7. The preliminary investigations were conducted directly from the field strain data, with conclusions regarding the static behaviour of the existing RC bridge. The maximum strains recorded in the longitudinal direction were +3.44 µε, +8.13 µε and +1.74 µε at mid-span 1 in the cross-sectional member of the girder in load cases 1, 2 and 3, respectively. Maximum tension strains of +17.10 µε, +21.42 µε and +19.41 µε were obtained from the girders of span 2 in various load cases. The maximum measured strains recorded on the beams of span 3 were +18.95 µε, +23.85 µε and +18.25 µε for each static load case. The largest strains occurring at mid-span 4 of the girders were +20.25 µε, +28.28 µε and +13.88 µε. All strains were multiplied by the Young’s modulus of the concrete material to obtain the stresses. The FFT results of accelerations in the vertical, horizontal and longitudinal directions were analysed to compute the natural frequencies, as displayed in Figure 8 and Figure 9. The first natural frequencies were determined through the FFT method, with 3.9 Hz, 4.09 Hz, 3.8 Hz and 3.1 Hz for the dynamic behaviour of spans 1, 2, 3 and 4, respectively. Note that the dynamic behaviours of the existing bridge were not used to update the FE modelling of the bridge structure in this study; the static measured strains of the girders of the structure were calibrated in the FE modelling by comparison with the computed data for every load case applied to the model.

### 3.4. FE Model Updating and Analysis

**Figure 10:**
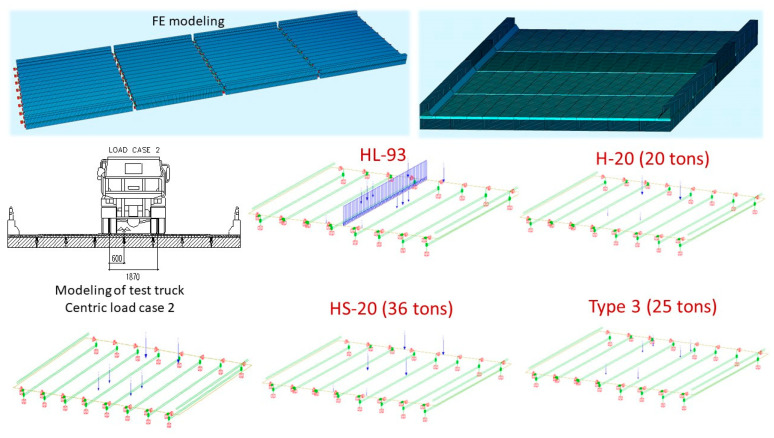
FE modelling of the RC bridge structure and modelling of truck configurations.

The FE modelling of the Vietnamese ThiThac bridge structure is built in SOFISTIK software. The main structural components of bridge modelling include 2-node frame elements (BEAM) to represent eight rectangular longitudinal girders, 4-node shell elements (QUAD) for the deck and spring elements to simulate elastic supports at bearing locations for boundary conditions, as shown in Figure 10. The model is developed so that the configurations of the load testing vehicle are reproduced in the model as the actual test truck on the bridge. The cross-sections of the girders (height: *h* and width: *b*) and the material properties of the concrete are assigned to the various structural elements in the model. Stiffness properties are selected to update the FE model, including the girder stiffness (*E_c_* and moment of inertia: *I*) and deck stiffness (thickness: *t_d_* and *E_c_*). Comparisons of strain values are made between analytical data and measured results. The initial FE model is updated by modifying the various cross-sectional girders, material properties and boundary conditions until the results match the measured strain responses in the field testing, so that the final updated FE model will be accepted with the average percentage error minimised, being less than 10%. Note that the stress values at the bottom of the girders that relate to the installed gauge positions are determined through the neutral axis (N/A), the moment of inertia calculations and the bending moments. All stresses are divided by the elastic modulus of the girders to obtain the strains in terms of micro-strain (µε), which can be used to compare the calculated strains in the updated FE model. The N/A locations are determined based on the cross-sectional properties of the rectangular girders and the transformed deck.

Table 1 contains the stiffness properties of the adjustable parameters, the lower and upper limits and also the initial and final values of the FE model update. The initial concrete elastic modulus (*E_c_*) is 25 GPa, the Poisson’s ratio is 0.25 and the density is 2500 kg/m^3^, assumed for girders, deck slabs and parapets according to EN 1992-1-1:2004. The initial height and width of the RC girders, and the average thickness (*t_d_*) of the concrete deck slab, are based on the design dimensions defined for the cross-sectional groups to assign to those girder members. The cross-sectional stiffness properties (*I_1_*, *I*_2_, *I*_3_, *I*_4_, *I*_5_, *I*_6_, *I*_7_, *I*_8_) of the RC girders are calibrated in the optimisation procedure. Limits in the cross-sectional properties of structural members are increased and decreased from the initial values of the height and width of the RC girder, as in the examples cited here [30].

**Figure 11:**
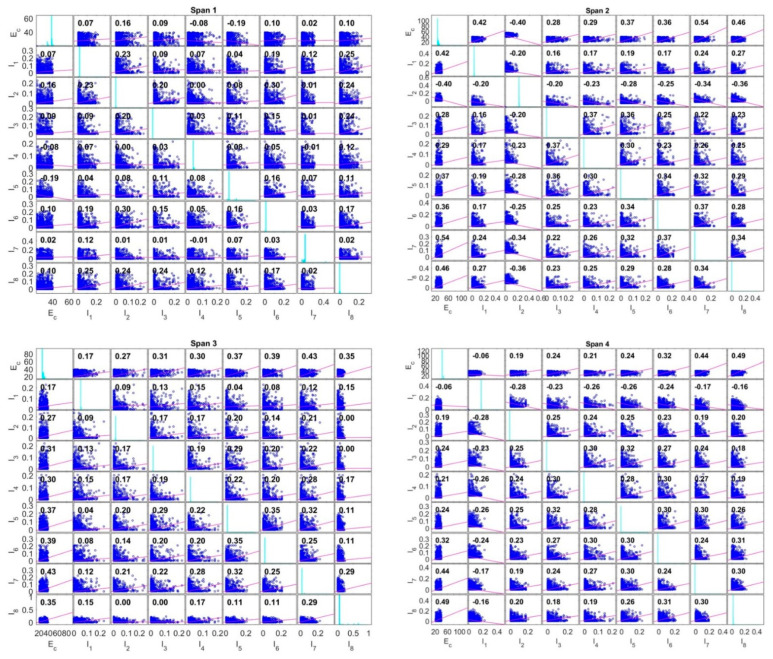
The results of the adjustment of the stiffness parameters for spans 1, 2, 3 and 4.

The strain comparison processing and the structural stiffness update procedure indicated the following results for each span. The concrete elastic modulus of the final calibrated span 1 increases by 10.52%, from 25 GPa to 27.63 GPa, while those of spans 2 and 3 decrease by 15.04% and 15.92%, respectively. The elastic modulus of span 2 climbs from 2.72% to 25.68 GPa, while the moment values of inertia from beam 2 to beam 7 drop considerably. Some exterior beams were not made to calibrate the measured data for the stiffnesses because the sensors were not mounted to these beams and the values were too small. Figure 11 displays the initial and final values of the parameters of the stiffness properties in the first span of the RC bridge structure. The variables of the FE model update in the optimisation procedure are extracted to plot all pairs of calibrated variables in the correlation coefficient matrix, with the corresponding labels with which the lower and upper bounds are illustrated in the diagrams. The results of the stiffness parameters indicated that the calculated data are more focused on the moment of inertia of the girders than on the concrete elastic modulus of the entire structural span. The optimisation process has been carried out by modifying the various height and width values of each girder in the span as independent variables, while the elastic modulus is used to obtain the same parameter for all components of the structure. The correlation coefficients of the stiffness parameters range from −1.0 to +1.0. These values are less than 0.5, indicating the weak linear relationship between the two stiffness variables. There are some negative values of the correlation coefficient that are reflected in the relative movements of the two stiffness variables, such as sloping downward or changing in the opposite direction. Some particular cases of the correlation coefficient matrix close to zero demonstrated that the two stiffness variables have little to no linear relationship and could be dependent.

**Figure 12:**
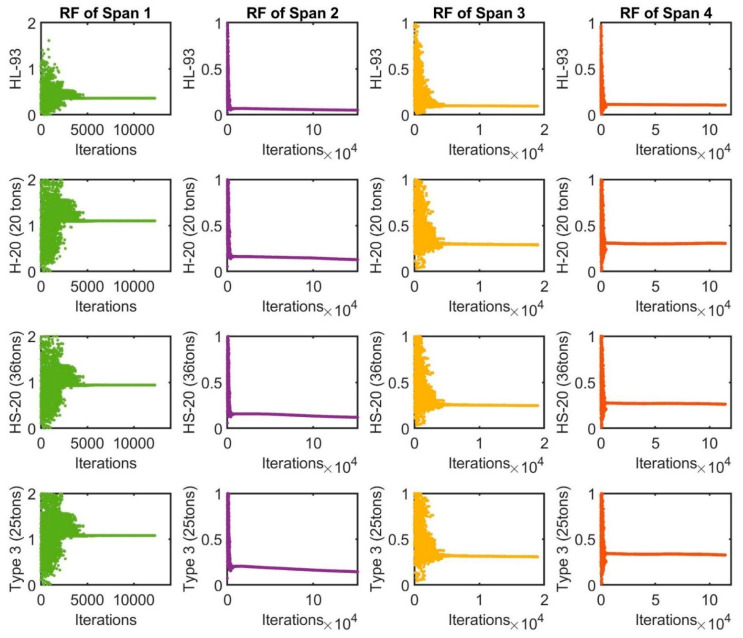
The rating factors of the truck load configurations according to the AASHTO design codes.

Figure 12 shows the *RF* values for each span of the bridge in the update procedures of the FE model, in which the rating factors of the bending stresses of the structural members were calculated by applying the truck load configurations according to the AASHTO standards in the final updated FE models. The spans of the bridge structure are very short spans that could not be applied to all load configurations. Some vehicle load configurations of design standards have been used for the final calibrated FE models, such as HL-93; H-20 (20 tons); HS-20 (36 tons) and Type 3 (25 tons). HL-93 is combined from the truck of 32.5 tons with a lane load of 9.3 kN/m and 22 tons of the vehicle with two axles, so that the *RF* values of all spans are less than 1.0. Span 2 is the shortest span and has the lowest *RF* of the entire bridge, with the *RF* values equal to 0.12, 0.11 and 0.14, corresponding to H-20, HS-20 and Type 3. The loads should decrease the actual capacity of the bridge by 2.40 tons, 3.96 tons and 3.50 tons, respectively. From the load rating results, spans 3 and 4 have *RF* values less than 1.0, indicating that these spans are critical for H-20, HS-20 and Type 3 loading. Span 1 was rated the best of all spans, with an *RF* value of approximately 1.0.

**Figure 13:**
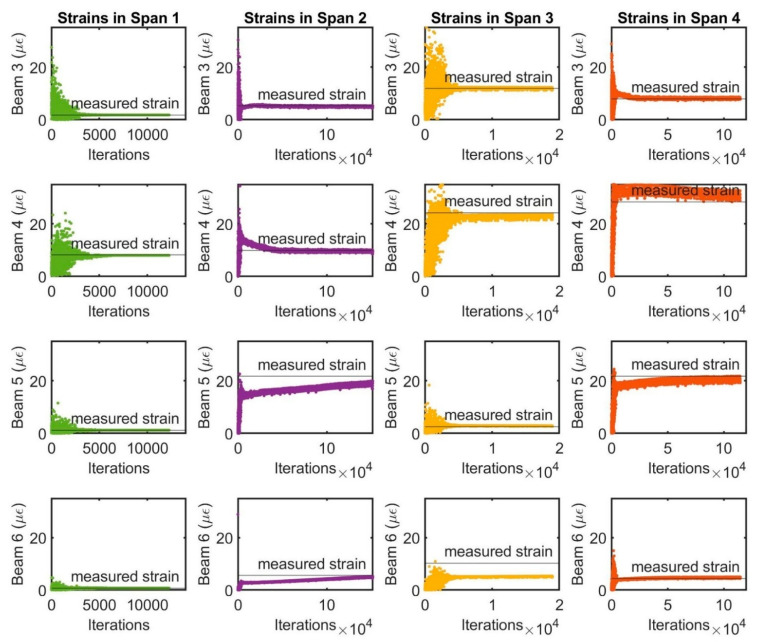
The results of numerical strain responses of beams 3, 4, 5 and 6 in the bridge spans.

The results of the computational strain responses of structural members under static centric load case 2, which was used to calibrate the FE model with the measured data in the field testing, are shown in Figure 13. The numerical comparison of the computed and measured strain historical responses has been used to determine the objective function of the average percent error during the calibration procedure. The measured strain records of spans 1, 2 and 4 are similar to the computed responses, while the strain value of beam 6 in span 3 is not very similar, and the member stiffness should be adjusted further because it could not be well represented. The measured and calculated strain on beam 4 of span 1 had the highest values of 8.15 µε and 8.00 µε, respectively, with the scale error of 1.84%. In span 2, the strains at the beam 5 were 21.67 µε for testing and 19.88 µε for computing, with the error of 8.26%. The error value of the strain on beam 4 of span 3 is 2.27%, compared to the measured and computed strain of 24.14 µε and 23.59 µε, respectively. With span 4, the error of strain at beam 4 is 5.67%, with 28.35 µε for field strain and 29.96 µε for the FE method.

**Figure 14:**
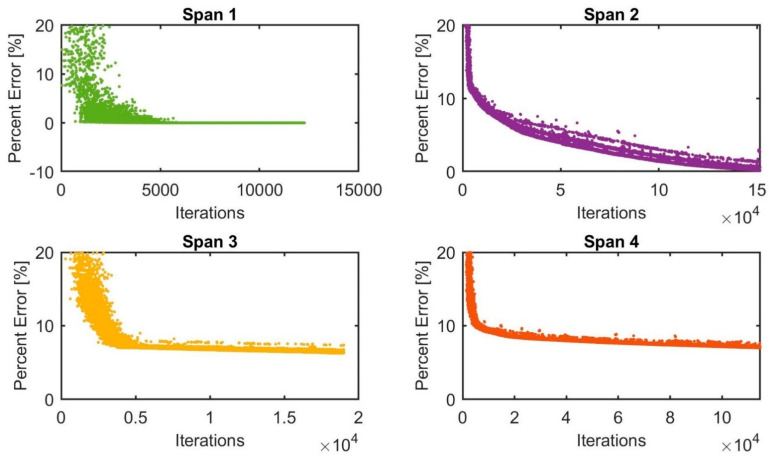
The percentage error values using the GA method for updating the FEM modelling of spans 1, 2, 3 and 4.

Figure 14 shows the percentage error of the GA algorithm for the FE model updating scenarios of all structural bridge spans with 12,285, 151,648, 19,004 and 114,695 iterations for spans 1, 2, 3 and 4, respectively. The GA algorithm converges rapidly and has almost the same convergence rate for all spans that start converging after 5000 iterations. The percentage error for span 1 is 0.01%, being the lowest, while span 4 has an error equal to 7.16%, which is the highest. Spans 2 and 3 have error values less than 10%, with a percentage error of 0.21% and 6.31%, respectively. A good representative model would generally have a percentage error value of less than 10%.

## 4. Conclusions

In this study, the update of the existing bridge was developed to obtain a calibrated field model that could accurately represent the actual responses of the bridge structure and could also apply any truck load configurations to compute realistic load ratings according to AASHTO design standards. For these main reasons, the analytical data in the FE model from the SOFISTIK software were generated automatically by interfacing with the MATLAB software to compare the measured data in the field testing, in order to produce the final updated model. The most important conclusions obtained from the final updated FE model of the RC bridge based on the field truck load testing are drawn as follows.

(1) Efforts were successful in updating the FE models of four RC bridge spans to reproduce the measured strain responses, with acceptable and accurate results.

(2) The final rating factors were calculated for stress responses using load configurations including HL-93; H-20 (20 tons); HS-20 (36 tons) and Type 3 (25 tons). Rating factors were calculated using the allowable stress method to predict the actual load limit of the structural members. The load rating factors of all spans were less than 1.0, indicating that this bridge was considered of a critical load to ensure structural safety and serviceability for repair and maintenance. In its current condition, this bridge rates very poorly according to the HL-93 and other specified load cases, in which the repair of the structure must be considered, as well as reducing the load capacity and serviceability limits.

(3) Useable strain transducers were installed on concrete surfaces to record the historical responses of the RC girders in the static load case, which was used to calibrate the numerical strains in the analytical FE model while minimising the error function. The percentage error values of all spans were less than 10%, while the error values between the numerical and measured strain responses at the maximum strain values of each span were below 10%.

(4) The stiffness properties of the main girders and the concrete deck slab were modified by the GA method. The stiffness values in spans 2, 3 and 4 were significantly reduced, while the stiffness parameters in span 1 were dramatically increased. Because the connections of structural members could be greatly reduced, the behaviour of all beams was sharply changed. These issues mean that the neutral axis locations of the RC girders have significant changes in the actual structure.

(5) There are some limitations in the proposed methods, related to the short spans of the RC bridge, which do not apply all load configurations according to the AASHTO standards. Some truck loads were built into the final updated FE model of the bridge to predict load ratings. To better overcome these issues, more sensors should be mounted on the girders, but this would sometimes be unnecessary, increasing the cost of field load testing. Furthermore, some structural spans are too short outside of the standard rating process related to truck axle dimensions in the design load configurations, which could be reduced so as to be applied in the updated FE modelling.

(6) In a future study, the updating of the FE bridge model could be developed by using the experimental dynamic and static responses of the structure. This proposed approach could be used to build various construction systems with different inputs and materials.

## Figures and Tables

**Table 1:** The results of FE model updating for initial and final values of variable parameters.

Stiffness Parameters	Initial Value	Lower Limit	Upper Limit	Final Values			
				Span 1	Span 2	Span 3	Span 4
*E_c_*, [GPa]	25	21	40	27.63	21.24	21.02	25.68
*t_d_* [mm]	100	-	-	-	-	-	-
*h* [mm]	400	0.15 × *h*	2.5 × *h*	-	-	-	-
*b* [mm]	1180	0.15 × *b*	2.5 × *b*	-	-	-	-
*I*_1_, [m^4^]	6.29 × 10^−3^	3.18 × 10^−6^	0.24	12.89 × 10^−3^	12.23 × 10^−3^	22.49 × 10^−3^	6.782 × 10^−3^
*I*_2_, [m^4^]	6.29 × 10^−3^	3.18 × 10^−6^	0.24	4.891 × 10^−3^	70.08 × 10^−3^	3.974 × 10^−3^	0.030 × 10^−3^
*I*_3_, [m^4^]	6.29 × 10^−3^	3.18 × 10^−6^	0.24	0.754 × 10^−3^	0.042 × 10^−3^	4.413 × 10^−3^	0.123 × 10^−3^
*I*_4_, [m^4^]	6.29 × 10^−3^	3.18 × 10^−6^	0.24	25.73 × 10^−3^	0.006 × 10^−3^	3.236 × 10^−3^	0.943 × 10^−3^
*I*_5_, [m^4^]	6.29 × 10^−3^	3.18 × 10^−6^	0.24	12.74 × 10^−3^	0.076 × 10^−3^	0.928 × 10^−3^	1.545 × 10^−3^
*I*_6_, [m^4^]	6.29 × 10^−3^	3.18 × 10^−6^	0.24	10.79 × 10^−3^	0.021 × 10^−3^	2.712 × 10^−3^	0.607 × 10^−3^
*I*_7_, [m^4^]	6.29 × 10^−3^	3.18 × 10^−6^	0.24	29.14 × 10^−3^	0.850 × 10^−3^	0.027 × 10^−3^	1.959 × 10^−3^
*I*_8_, [m^4^]	6.29 × 10^−3^	3.18 × 10^−6^	0.24	5.467 × 10^−3^	0.025 × 10^−3^	4.531 × 10^−3^	11.69 × 10^−3^
Percent Error [%]	-	-	-	0.01	0.21	6.31	7.16
*RF* (HL93)	-	-	-	0.36	0.05	0.09	0.10
*RF* (H-20, 20 tons)	-	-	-	1.09	0.12	0.28	0.31
*RF* (HS-20, 36 tons)	-	-	-	0.94	0.11	0.24	0.26
*RF* (Type 3, 25 tons)	-	-	-	1.07	0.14	0.30	0.32

## Data Availability

The data is available from the corresponding author (Duc Cong Nguyen, Email: cong.nguyen@polsl.pl and nguyencongduc@muce.edu.vn) upon reasonable request.
